# Insights into the antimicrobial effects of ceritinib against *Staphylococcus aureus *in vitro and in vivo by cell membrane disruption

**DOI:** 10.1186/s13568-022-01492-w

**Published:** 2022-11-28

**Authors:** Shasha Liu, Pengfei She, Zehao Li, Yimin Li, Yifan Yang, Linhui Li, Linying Zhou, Yong Wu

**Affiliations:** 1 Department of Laboratory Medicine, The Third Xiangya Hospital, Central South University, Changsha, 410000 Hunan China; 2 Department of Laboratory Medicine, The Affiliated Changsha Hospital of Xiangya School of Medicine, Central South University, Changsha, 410000 Hunan China

**Keywords:** Drug repurposing, Ceritinib, Methicillin-resistant *Staphylococcus aureus*, Biofilm, Persister

## Abstract

According to a 2019 report from the Centers of Disease Control and Prevention (CDC), methicillin-resistant *Staphylococcus aureus* (MRSA) was listed as one of the “serious threats” that had become a global public challenge in hospitals and community. Biofilm-associated infections and refractory persisters of *S. aureus* also impede the effectiveness of conventional antibiotics that have greatly increased difficulty in clinical therapy. There is an urgent need to develop new antimicrobials with antibiofilm and anti-persister capacities, and drug repurposing is the most effective and most economical solution to the problem. The present study profiles the antimicrobial activity of ceritinib, a tyrosine kinase inhibitor, against *S. aureus *in vitro and in vivo. We investigated the antimicrobial efficacy of ceritinib against planktonic and persistent *S. aureus* by a time-killing kinetics assay. Then, antibiofilm effect of ceritinib was assessed by crystal violet staining and laser confocal microscope observation. Ceritinib showed biofilm inhibition and mature biofilm eradication, and possesses robust bactericidal activity against *S. aureus* persisters. We also evaluated antimicrobial efficacy in vivo using a subcutaneous abscess infection model. Ceritinib ameliorated infection in a subcutaneous abscess mouse model and only showed negligible systemic toxicity in vivo. Mechanism exploration was conducted by transmission electron microscopy, fluorescently labeled giant unilamellar vesicle assays, and a series of fluorescent dyes. In conclusion, we find ceritinib represents potential bactericidal activity against MRSA by disrupting cell membrane integrity and inducing reactive oxygen species production, suggesting ceritinib has the potential to treat MRSA-related infections.

**Supplementary Information:**

The online version contains supplementary material available at 10.1186/s13568-022-01492-w.

## Key points

- Ceritinib showed fierce lethality to the planktonic and persistent *S. aureus*.
- Ceritinib were highly effective in MRSA biofilm eradication and inhibition.
- The bactericidal mechanisms of ceritinib were showed by membrane breaking and ROS activation.

**Supplementary Information:**

The online version contains supplementary material available at 10.1186/s13568-022-01492-w.

## Introduction

*Staphylococcus aureus*, a human symbiotic and pathogenic bacterium that mainly colonizes the skin and mucosa, is one of the primary causes of hospital- and community-acquired infections (Lakhundi and Zhang 2018; Tan et al. 2020). *S. aureus* poses severe threats to individuals, such as bacteremia, subcutaneous abscess, toxic shock syndrome, and endocarditis, which increases the burden on social economy and decreases quality of life (Guo et al. 2020; Hodille et al. 2017). According to a 2019 report from the Centers of Disease Control and Prevention (CDC), more than 2.8 million antimicrobial-resistant infections occur each year in the USA, resulting in over 35,000 deaths. And the CDC considers 11 bacteria as “serious threats”, including methicillin-resistant *Staphylococcus aureus* (MRSA) (Regen 2021). MRSA has been experiencing explosive growth due to the rapidity of resistance progress and the shortage of infection management. It’s estimated more than 90% *S. aureus* is resistance to penicillin and cause more than 80,000 invasive infections in the United States (David and Daum 2010; Lakhundi and Zhang 2018). In worldwide, exceed 53 million people may be colonized with MRSA in 2011 (Peacock and Paterson 2015). MRSA wreaks havoc in hospitals and throughout communities, and poses a global public challenge (Lakhundi and Zhang 2018).

Apart from the tolerance to almost all β-lactam antibiotics mediated by resistant mutations, *S. aureus* has two other primary mechanisms to resist the bactericidal effectiveness of conventional antibiotics. Establishing defensive biofilms that form a shielded polymeric extracellular matrix is of great importance to antibiotic and immunological protection (Pinto et al. 2019). Biofilm-associated infections are often related to the usage of interventional devices, such as implanted catheters and prosthetic heart valves. Not only can biofilms restrict the transport of antimicrobials through specific binding to extracellular polymeric substances, electrostatic interactions, and enzymatic degradation, but they can also maintain a substantially different physiological status than planktonic cells can. As a result, the bacterial cells in the biofilms are protected from adverse conditions and hostile host attack and hence aggrandize the complexity of antimicrobial resistance (Craft et al. 2019). Additionally, as a nongrowing dormant phenotype, persisters also tolerate lethal antibiotics and are primarily relevant to recalcitrant and recurrent complications of biofilm-associated infections (Conlon et al. 2016; de Breij et al. 2018). Although conventional first-line antibiotics such as ciprofloxacin (CIP), vancomycin (VAN), and rifampicin (RFP) have powerful effects against planktonic bacteria, they are invalid against the persister phenotype (Yu et al. 2021). Therefore, the existence of biofilms and persisters is a formidable task for health care systems and human beings. With the emergence of MRSA, especially vancomycin-resistant MRSA, and the discovery of vancomycin-resistant β-lactam drugs that can induce MRSA, it is apparently urgent to find new antimicrobial drugs to deal with refractory resistant pathogens (De Oliveira et al. 2020). Our research aims for overcoming antimicrobial resistance has been based on the theory that antimicrobial agents that promote membrane disruption are prone to be kill bacteria, rather than develop drug resistance (Regen 2021). Specifically, if antimicrobial agents contribute to the way of destroying the plasma membrane integrity, the common mechanisms of drug resistance would be circumvented, such as enzymatic degradation and efflux pump, and so on (Regen 2021; Steinbuch and Fridman 2016).

As an alternative strategy, drug repurposing means to discovery existing or pre-clinical drugs for new therapeutic opportunities. It has several advantages. First, the details of chemicals, such as structure, pharmacological properties and safety profiles, was already clear. Second, it has practical economic advantages. Third, repurposing drugs can offer new pathways or targets to study new perspectives for curing diseases (Peyclit et al. 2019).

Ceritinib (CERI), also called LDK378, is a tyrosine kinase inhibitor of anaplastic lymphoma kinase (ALK) approved by the Food and Drug Administration (FDA) in 2014.(Shaw et al. 2014) It shows impressive activity in ALK-rearranged non-small-cell lung cancer and is simultaneously inhibited by downregulating the signal effector YB1 (Crunkhorn 2017). In 2019, some scholars found that CERI is effective against *Mycobacterium tuberculosis* strains and elucidated satisfactory activity in a BALB/c mouse model (Liu et al. 2019). Although CERI has remarkable effectiveness in cancer treatment, bacteria-related studies as an antimicrobial against *S. aureus* are few and far between.

Here, we aimed to explore the antibacterial activity and possible mechanism of CERI against *S. aureus* and further determine the antibiofilm and anti-persister effects of CERI. Meanwhile, we investigated the effectiveness in a subcutaneous abscess mouse model and the toxicity in vivo.

## Materials and methods

### Bacterial strains, medium and reagents

*Staphylococcus aureus* ATCC 43300, Newman, and RJ-2 were kindly provided by Min Li (Shanghai Jiaotong University, Shanghai, China). SAJ1 and other clinical isolates were collected in the Third Xiangya Hospital of Central South University (Changsha, China) (She et al. 2020). In addition, gram-negative bacteria, including *Acinetobacter baumannii* ATCC 19606, *Klebsiella pneumoniae* ATCC 700603, and *Escherichia coli* ATCC 25922, and gram-positive bacteria, including *Enterococcus faecalis* ATCC 29212 and *S. aureus* ATCC 29213, were provided by Juncai Luo (Tiandiren Biotech). *Pseudomonas aeruginosa* PAO1 was obtained from the Mingqiang Qiao research group (Nankai University). *Enterococcus faecalis* ATCC 29212 was grown in brain–heart infusion (BHI, pH 7.2 ~ 7.6) broth. Other Gram-positive and Gram-negative strains were cultured in soybean trypsin broth (TSB, pH 7.3) and Luria–Bertani broth (LB, pH7.2) medium, respectively. All medium were purchased from Solarbio (Shanghai, China) and sterilized by autoclaving at 121 ℃ for 15 min. All bacteria were propagated at 37 °C with shaking at 200 rpm. CERI and other chemicals were purchased from MedChem Express (New Jersey, the United States) or Sigma Aladdin (Shanghai, China). For antibacterial test in vitro and in vivo, ceritinib was prepared in dimethyl sulfoxide (DMSO) and cremophor EL/ethyl alcohol mixture (1:1, v/v), respectively.

### Antimicrobial susceptibility testing

According to the Clinical & Laboratory Standards Institute (CLSI) guidelines, a series of dilutions of antimicrobials in Mueller–Hinton (MH, pH 7.0 ~ 7.4) broth were added to a 96-well microplate. Bacterial suspension was prepared by adjusting to 0.5 McFarland (McF) and diluting with 1:100, which was corresponding approximately 1.5 × 10^6^ CFU/mL. Then, an equal volume of MH broth containing log phase bacterial cells was added to the wells and the bacterial suspension was grown at 37 °C for 16 h, except for oxacillin and VAN, which were grown for 24 h. Then, the minimum inhibitory concentration (MIC) was determined by no visually discernible turbidity in the wells. In addition, 10 μL of bacterial suspension from the well of 1 × MIC was further spread on blood agar plates incubating at 37 ℃ for 24 h. The minimum bactericidal concentration (MBC) was determined by the lack of observable colony growth on plates (Dos Santos et al. 2020).

### High throughput screening

To identified potential antimicrobial compounds against *S. aureus* strains, the molecular library was screened. These compounds were stored in dimethyl sulfoxide (DMSO) at 10 mM with 30 μL of primary stock solutions a − 20 °C. In initial screening process, bacterial suspension was prepared by adjusting to 0.5 McFarland (McF) in MH broth medium when grown to until mid-logarithmic phase and transferred to 96-well plates with 99 μL/well. After add 1 μL stock solution of individual chemicals into each well (corresponding 100 μM final concentration), the turbidity was measured at 600 nm optical density (OD) to identified the growth condition of bacteria. In second screening process, we reduce the concentration of each chemical (50 μM) to refine candidates. The culture condition is at 37 ℃ with 5% CO_2_ humid surroundings. The OD_600_ was measured after 16 h incubation at 37 °C. All compounds were screen for three repetitions. Excluded antimicrobials, six hits were determined (Additional file 1: Table S1). In the Final process, the MIC and MBC were measured, and one hit (ceritinib) was selected for further investigation (Li et al. 2020).

### Time growth and killing kinetics assay

The cultures of *S. aureus* strains were adjusted to 0.5 McF in fresh TSB broth from overnight shaking at 37 °C and 180 rpm. The cells were diluted 1:100 in TSB to a final cell concentration of ~ 1 × 10^6^ CFU/mL in the presence of 1/4 to 4 × MIC of CERI and incubated at 37 °C and 180 rpm. At the time points of 0, 2, 4, 8, 12 and 24 h, an aliquot of the bacterial suspension was taken and measured the turbidity by detecting OD at 630 nm, and colony-forming units (CFUs) counting. For CFU counting, the bacterial suspension was serially diluted tenfold with saline and spotted on blood agar. After incubation overnight, the CFU were counted (De Oliveira et al. 2020; Gaurav et al. 2021).

### Anti-biofilm activity of CERI

Following a previously reported procedure (Le et al. 2020) for the biofilm inhibition assay, a single colony was transferred into TSB and grown overnight at 37 °C and 180 rpm. The bacterial suspension was treated with CERI in a series of twofold dilutions prior to inoculation in a 96-well plate. After 24 h of incubation, the supernatant was removed, and the biofilms were washed with sterile saline to remove the planktonic cells. Then, 200 µL of 0.25% crystal violet was added to each well and dyed for 15 min. After removal of the extra dye solution, the biofilm was washed again with saline and dissolved in 95% ethanol. The absorbance (A) was measured at 570 nm by a microplate spectrophotometer (Bio-Tek, USA). The experiment was performed in triplicate on two independent days.

To determine the biofilm eradication effects of CERI, overnight cultures of *S. aureus* ATCC 43,300 were diluted 1:100 with fresh TSB media, and 200 µL was added to each well in flat-bottom 96-well plates in the presence of 2% (wt/vol) glucose, which was reported to contribute to promoting biofilm formation (She et al. 2020). The plates were incubated at 37 ℃ for 24 h to form mature biofilms. After removing the supernatant, 200 µL of TSB broth in the presence of CERI or DMSO was added to each well. After 24 h of incubation, the supernatant was removed, and the biofilms were quantified by crystal violet staining as previously described.

After treatment with antimicrobials as described, the supernatant was removed, and 200 µL saline was added to each well. The biofilms were mixed adequately with tips, and 100 µL of the suspension was removed to perform CFU counting as described (Tan et al. 2019).

### Confocal laser scanning microscopy (CLSM)

The overnight culture was added to a 6-well cell culture plate (Corning Costar, Cambridge, MA, United States) containing serial concentrations of CERI or DMSO incubated statically with 18 mm × 18 mm sterile glass cover slides at 37 °C for 24 h. The biofilms formed on coverslips were stained with SYTO 9 (green) and PI (red) (Thermo Fisher Scientific, Shanghai, China) at a final concentration of 10 μM. After staining for 15 min in the dark and fixing on object slides, the coverslips were photographed by CLSM (Zeiss LSM 800, Jena, Germany). Picture processing and image quantization were completed by ImageJ software (Tan et al. 2019).

### Cellular membrane permeability assay

The mid-log growth phase of *S. aureus* ATCC 43300 or its later platform growth phase persister cells were rinsed with saline and resuspended in HEPES buffer (pH 7.2). After adjusting to OD_600_ = 0.05, SYTOX Green fluorescence dye was added to the suspension to a final concentration of 2 μM and incubated for 15 min in the dark. After treatment with a series of concentrations of antimicrobials, the intensity of the fluorescence was detected every 5 min for a total of 30 min with excitation (λ_ex_) and emission (λ_em_) wavelengths of 504 nm and 523 nm, respectively (She et al. 2020).

### Cytoplasmic membrane depolarization assessment

Cytomembrane depolarization was evaluated by using DiSC3(5) fluorescent dye as described previously with minor modifications (Bai et al. 2021). In summary, the mid-log growth phase of *S. aureus* ATCC 43,300 was collected by centrifugation at 4 ℃ and 4000 rpm for 8 min and washed twice with HEPES buffer (5 mM, pH 7.2) with 5 mM glucose and 100 mM KCl solution. The bacterial suspension was resuspended in buffer to an OD_600_ of 0.2 and then incubated with DiSC3(5) (final concentration of 2 μM) for 45 min in the dark. Next, the above mixture was transferred into a 96-well black plate in the presence of serially diluted concentrations of CERI. Fluorescence intensity changes were captured every 30 s for a total of 5 min by a microplate reader (λ_ex_ = 622 nm, λ_em_ = 670 nm). Melittin was used as a positive control, and DMSO was used as a negative control.

### Propidium iodide (PI) uptake assay

The acquisition of bacterial sediment followed the same process as above. Then, 10 μL of PI was added to 5 mL of bacterial suspension for a final concentration of 10 μM. After incubation for 30 min at 37 °C, the uncombined dye was removed by washing with PBS, CERI was added to each well, and the fluorescence intensity was recorded at λ_ex_ = 535 nm and λ_em_ = 617 nm.

### Reactive oxygen species (ROS) qualification

According to the operation manual, ROS were detected by a 2′,7′-dichlorofluorescein diacetate (H2DCFDA) probe. Briefly, the overnight growth of *S. aureus* ATCC 43300 was washed and suspended in PBS to OD_600_ = 0.5. The bacterial suspension was incubated with H2DCFDA (10 μM) for 30 min in the dark. After washing twice with PBS, 10 μL of CERI was added to 90 μL of the prepared bacterial suspension. The fluorescence intensity was monitored by an automatic microplate reader at λ_ex_ = 488 nm and λ_em_ = 525 nm.

### Membrane fluidity assessment

The overnight culture of *S. aureus* ATCC 43,300 was diluted 1:1000 in fresh TSB media and incubated to OD_600_ = 0.5. The Laurdan liquid dye (Gunther et al.; Kim et al. 2019) was added (final concentration to 10 μM) and incubated for 10 min at room temperature. After centrifuging at 4 ℃ and 4000 rpm for 8 min, the supernatant was removed, and the pellet sediment was washed three times with PBS. After treatment with CERI, daptomycin or DMSO, the fluorescence intensities of fivefold enrichment were determined at λ_ex_ = 350 and λ_em_ = 440 nm and 490 nm. The generalized polarization (GP) values of Laurdan were calculated as follows (Hyun et al. 2020):

$$\mathrm{GP}=\frac{{I}_{440}-{I}_{490}}{{I}_{440}+{I}_{490}}$$

## Intracellular and extracellular ATP measurement

As described by our previously reported article (Liu et al. 2021). The ATP release of *S. aureus* ATCC 43300 was measured by an Enhanced ATP Assay Kit (Beyotime, Shanghai, China). Briefly, overnight cultured pellets were resuspended in PBS and adjusted to OD_600_ = 0.5. After treatment with CERI (0 ~ 32 μg/mL) for 1 h, the bacterial suspension was centrifuged at 4 ℃, and the level of extracellular ATP was measured according to the operation manual. In addition, part of the bacterial sediments was collected and fractured by a prepared lysis solution and further used for intracellular ATP determination.

### Fluorescently labeled giant unilamellar vesicles (GUV) assay

The vesicle suspension consisting of DOPC/DOPG (7:3) labeled with 2% (w/w) FITC was induced by sedimentation in a 110 mM glucose solution diluted slowly by 1:30. After centrifugation at 4 ℃ and 3000 rpm for 2.5 min, the sediment was collected and diluted 1:3 in sucrose/glucose commixture (1:6). Eighteen microliters of the prepared GUV suspension were mixed with 2 × MIC CERI for 15 min. The GUVs were observed and imaged by CLSM (Zeiss LSM 800, Jena, Germany) (Kim et al. 2019).

### Ultrastructure observation by scanning and transmission electron microscopy (SEM/TEM)

The strain of *S. aureus* ATCC 43300 was cultured overnight in TSB broth, and 10 μL aliquots were transferred into 20 mL fresh TSB. The pellets were incubated ~ 4 h to log phase and centrifuged at 4 ℃ and 4000 rpm for 8 min. The precipitate was washed with PBS and resuspended in TSB broth in the presence of 5 × MIC of CERI or DMSO. After incubation at 37 ℃ for 1 h, the bacteria were collected by centrifugation at 4 ℃ and 4000 rpm for 8 min. The samples were fixed with 1 mL glutaraldehyde and washed in 0.1 M cacodylate buffer. After being postfixed with 1% osmium tetroxide/1.5% potassium ferrocyanide for 1 h, the fixed cells were washed and incubated in a maleate buffer containing 1% uranyl acetate for 1 h. Then, the cells were washed twice in water, dehydrated in an alcohol gradient series, incubated in propylene oxide for 1 h, embedded in a 1:1 mixture of propylene oxide and Spurr’s low viscosity resin and polymerized at 60 ℃ for 48 h. Ultrathin sections (approximately 60 nm) were cut on a Reichert Ultracut-S microtome, picked up onto copper grids, and stained with lead citrate. Samples were imaged by SEM and TEM (HITACHI, Tokyo, Japan) (She et al. 2020).

### Persister time-killing assay

To form persisters of *S. aureus* strains, the culture was prepared at 37 °C with shaking at 200 rpm for 24 h, washed three times with PBS, and adjusted to OD_600_ = 0.2. A 1–8 × MIC CERI (10 × MIC VAN and CIP as control) was added, and CFU counting was performed as described above at the time points of 0, 2, 4, 6, and 8 h (Liu et al. 2021).

### Hemolysis assay

Human red blood cells (RBCs) were purchased from Hemo Pharmaceutical and Biological Co. (Shanghai, China). The RBCs were centrifuged at room temperature, diluted with PBS and transferred into a 96-well plate to a final concentration of 5% (vol/vol). Then, CERI was added to each well at a concentration of 1–32 μg/mL. After incubation at 37 °C for 1 h, the supernatant was removed, and hemolysis was measured at A570 nm. 0.1% Triton X-100 and 1% DMSO were set as positive (Pos.) and negative (Neg.) controls, respectively. The hemolysis rate was calculated as follows:

$$\mathrm{Hemolysis }\left({\%}\right)=\frac{{A}_{sample}-{A}_{Neg.}}{{A}_{Pos.}-{A}_{Neg.}}\times 100\%$$

### Cytotoxicity assessment

To further evaluate the cytotoxicity of CERI in vitro to HK-2 (epithelial cells from human renal proximal convoluted tubule) and the 786-O cell line (human renal carcinoma cells) by Cell Counting Kit-8 (CCK-8, DojinDo, Japan), HK-2 cells were cultured in RPMI 1640 medium, and 786-O cells were grown in Dulbecco’s modified Eagle’s medium (DMEM). Both cell lines were supplied with 10% fetal bovine serum (FBS) (Jia et al. 2019; Zheng et al. 2015). Subsequently, 100 μL of cells was added to a 96-well plate at a final concentration of 5 × 10^3^ cells/well and incubated at 37 °C with 5% CO_2_ for 24 h for the sake of cell adhesion. After exposure to different concentrations of CERI or 0.1% DMSO, the cells were incubated in a normal tissue culture environment for 8 h (Zhou et al. 2020). The formula of the counting cell viability rate was calculated as follows:

$$\mathrm{Viability }\left({\%}\right)=\frac{{A}_{sample}-{A}_{blank}}{{A}_{0.1\%DMSO}-{A}_{blank}}\times 100\%$$

### Mouse cutaneous abscess model

All murine-related laboratory procedures were approved by the Ethics Committee of the Third Xiangya Hospital of Central South University (No. 2021sydw0245). The model was established as described in previous articles, with minor modifications (She et al. 2020; Zhang et al. 2021). Briefly, 7-week-old female, specific pathogen-free outbred ICR mice were randomly divided into two groups: (1) Vehicle group, (2) CERI-treated group. Each group had six mice. CERI was prepared in a Cremophor EL/ethyl alcohol mixture (1:1, v/v) as the storage concentration. Overnight cultured MRSA ATCC 43300 was washed and resuspended in saline to the concentration of 3 McF. Each mouse was injected s.c. with 0.5 mL of the bacterial suspension. At half an hour post-infection, a single dose of compounds (20 mg/kg) was administered (s.c.) every 12 h for a total of 3 days. The mice were euthanized by cervical dislocation on the last day, and the area of abscess was calculated with a caliper. Meanwhile, the infected skin was dissected and homogenized for bacterial load counting. Subsequently, the bacterial suspension was serially diluted tenfold with saline and spotted on blood agar. After incubation overnight, the CFU were counted.

### Histopathological analysis

To assess the degree of tissue inflammation in the skin abscesses and the systemic toxicity in vivo, the mice were sacrificed at the end point on Day 3, and the cutaneous tissues or organs (heart, liver, spleen, lung, kidney) were cut and fixed in 5 mL 4% neutral paraformaldehyde fixator solution (Servicebio, Wuhan, China) before hematoxylin and eosin (H&E) staining. H&E staining was captured by microscopy at random locations.

### Organ functional biomarker monitoring

At the end point on Day 3, after anesthesia, mouse blood was collected into EP tubes with or without EDTA-K2 for serum- and whole blood-related biomarker determination, respectively. The blood cell count and hematology parameters were determined by BC-5000vet hematology analyzer (Mindray, Shenzheng, China), which included white blood cell counting (WBC), red blood cell counting (RBC), platelet counting (PLT), and hemoglobin quantification (HGB). Then, the serum of the blood samples was obtained by centrifuging at 4 ℃ and 3000*g* for 15 min, and the chemical parameters, including alanine transaminase (ALT), blood urea nitrogen (BUN), and creatine kinase (CK), were measured by Labospect 003 automatic biochemical analyzer (Hitachi, Japan).

### Statistical analysis

The data were analyzed using Prism 9.0 (GraphPad Software, San Diego, CA, United States). All the data are presented as the mean ± standard deviation (SD), and statistical significance was analyzed by Student’s t test and one-way ANOVA. Differences between groups were considered significant when the *p* value was less than 0.05 (*P < 0.05, **P < 0.01, ***P < 0.001).

## Results

### CERI shows potent bactericidal activity against *Staphylococcus aureus*

Based on high throughput screening consisted of initial, secondary, and final screening sessions (Additional file 2: Fig. S1A), we chose CERI for further study. The chemical structural formula of CERI was shown in Additional file 2: Fig. S1B. We explored the antibacterial activities of CERI against gram-positive and gram-negative bacteria. CERI displayed potent antibacterial effects against both MRSA and methicillin-susceptible *Staphylococcus aureus* (MSSA) with MICs and MBCs of 8–16 μg/mL and 16–64 μg/mL, respectively. As we expected, CERI also exhibited identical activities against clinical MRSA isolates with MICs of 8–16 μg/mL. In addition, CERI also showed effective antimicrobial activity against *E. faecalis* ATCC 29,212 with an MIC of 16 μg/mL. However, there were no effects on gram-negative strains, whose MICs and MBCs exceeded 128 μg/mL (Table 1). In the light of the results, we chose the *S. aureus* strain for further investigation.

**Table 1:** Antimicrobial susceptibility of CERI, VAN, OXA and daptomycin against Gram-positive and Gram-negative pathogens

Strain	CERI (μg/ml)/(μM)		VAN (μg/ml)		DAP (μg/ml)		OXA (μg/mL)	
	MIC	MBC	MIC	MBC	MIC	MBC	MIC	MBC
*S. aureus*								
ATCC 25923	16/28.67	32/57.33	1	1	1	4	0.5	2
ATCC 29213	16/28.67	64/114.67	2	4	0.5	1	0.25	2
ATCC 43300	16/28.67	32/57.33	2	4	0.5	1	32	32
Newman	16/28.67	32/57.33	2	1	1	2	< 0.125	2
SAJ1	16/28.67	32/57.33	4	4	2	2	> 64	> 64
LZB1	16/28.67	16/28.67	1	2	0.5	1	0.25	0.5
RJ-2	16/28.67	32/57.33	1	2	0.5	1	8	32
USA 300	> 128/ > 229.33	> 128/ > 229.33	2	8	0.5	8	16	16
SA0524	16/28.67	16/28.67	2	8	1	1	8	8
SA2231	16/28.67	16/28.67	2	2	0.5	1	8	8
*E. faecalis*								
ATCC 29212	16/28.67	> 128/ > 229.33	4	16	4	8	–	–
*E. coli*								
ATCC 25922	> 128/ > 229.33	> 128/ > 229.33	> 128	> 128	> 128	> 128	–	–
*K. pneumoniae*								
ATCC 700603	> 128/ > 229.33	> 128/ > 229.33	> 128	> 128	> 128	> 128	–	–
*A. baumannii*								
ATCC 1195	> 128/ > 229.33	> 128/ > 229.33	> 128	> 128	> 128	> 128	–	–
MDR 1208	> 128/ > 229.33	> 128/ > 229.33	> 128	> 128	> 128	> 128	–	–
MDR 1069	> 128/ > 229.33	> 128/ > 229.33	> 128	> 128	> 128	> 128	–	–
*P. aeruginosa*								
PAO1	> 128/ > 229.33	> 128/ > 229.33	> 128	> 128	> 128	> 128	–	–

CERI started to inhibit bacterial cell growth effectively at a concentration of 1/2 × MIC (Additional file 2: Fig. S1C). Moreover, we found that CERI held commendable bactericidal efficacy with a concentration of greater than or equal to 2 × MIC to reach the limit of detection (LOD) within 4 h in a dose-dependent manner (Additional file 2: Fig. S1D). The data of in vitro bactericidal potential of plate images for the antimicrobial activity against *S. aureus* ATCC 43,300 was shown in Additional file 2: Fig. S1E.

### CERI inhibits biofilm formation and eradicates preformed biofilm

**Fig. 1:**
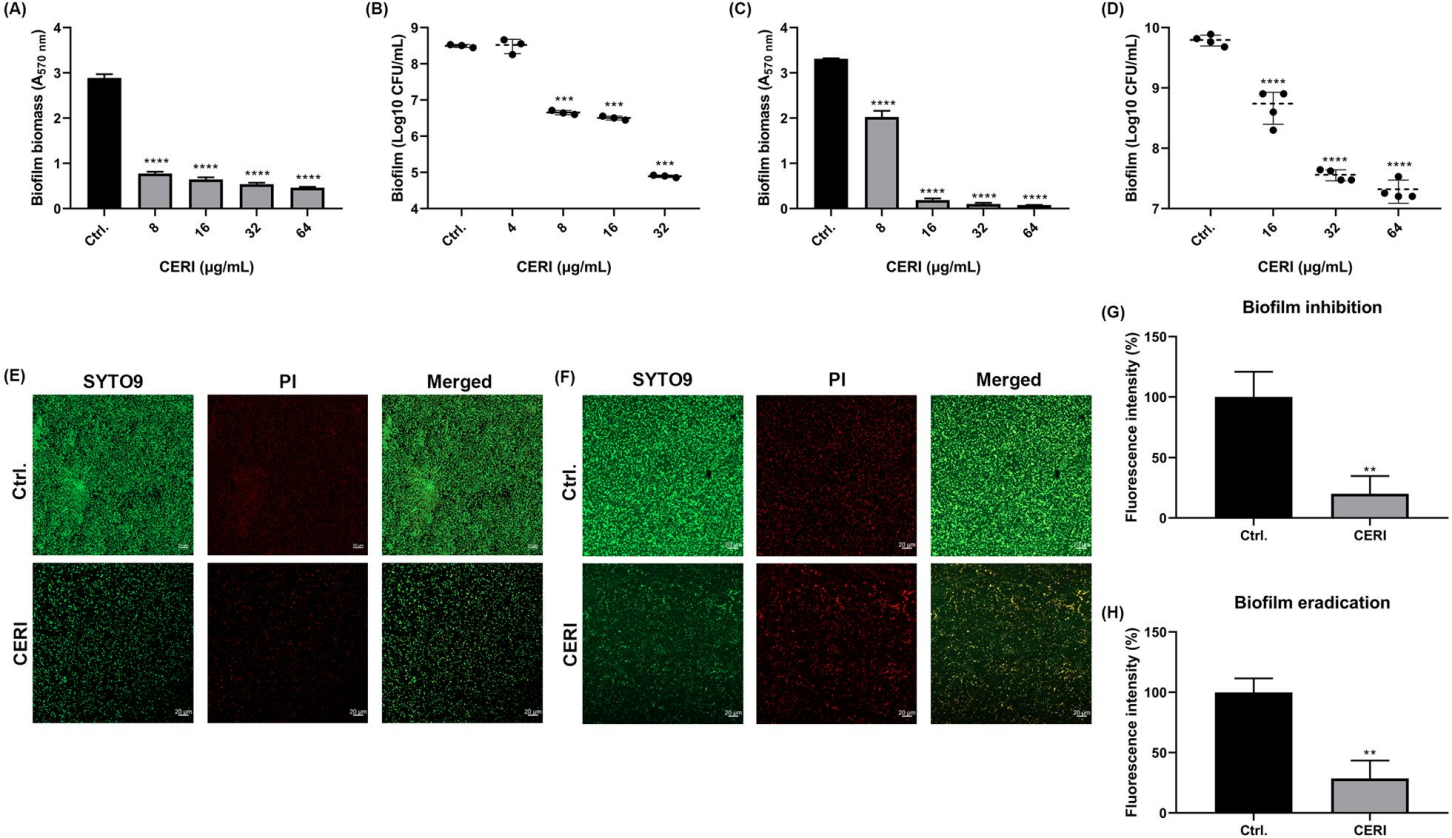
Anti-biofilm effects of CERI against *S. aureus*. Dose-dependent biofilm inhibition effect of CERI was determined using 0.25% crystal violet staining (**A**) and viable cell counting assay (**B**) after exposure for 24 h. Dose-dependent biofilm eradication effect of CERI was determined using 0.25% crystal violet staining (**C**) and viable cell counting assay (**D**) after forming mature biofilm and exposure for 24 h. STYO 9 and PI staining of MRSA ATCC 43300 in biofilm inhibition (**E**) and eradication (**F**) were observed by CLSM (×40) after exposure in CERI or DMSO for 30 min. Scale bars, 20 μm. Fluorescence intensity quantification analysis of the total biofilm biomass of biofilm inhibition (**G**) and biofilm eradication (**H**). The experiments were carried out in three biological repeats, and results correspond to average ± SD. The data were analyzed using one-way ANOVA with Dunnett’s multiple comparison test. Results were considered highly significant when ***P < 0.001 and ****P < 0.0001

The crystal violet (CV) staining method was one of the most used in vitro biofilm-associated experiments, enables quantification of total biofilm biomass consisting of live/dead cells and extracellular matrix (Magana et al. 2018). CERI displayed considerable biofilm formation inhibition activity against *S. aureus* ATCC 43,300 with reduced biofilm total biomass formation at a concentration of 8 μg/mL (Fig. 1A). Similarly, based on the viable cell counting assay, CERI also showed effective biofilm inhibiting effects against *S. aureus* at a concentration of 8 μg/mL in a dose-independent manner by decreasing the viable cells in the biofilms (Fig. 1B). For biofilm eradication, CERI disrupted biofilms at a concentration of 8 μg/mL, and biofilms were rare at a concentration of 16 μg/mL, as shown by crystal violet staining (Fig. 1C). Likewise, 32 μg/mL CERI notably reduced viable cells in the preformed biofilm by more than 2 log10 (Fig. 1D). That was to say, a significant reduction was observed by quantified with crystal violet assay (Fig. 1A and C), while less pronounced effect was observed with cell counting methods (Fig. 1B and D), suggesting CERI eradicated biofilms by decreasing the expression of extracellular matrix in biofilms.

SYTO 9 was a fluorescent permeable nucleic acid stain that was emitted a green fluorescent light in both live and dead cells. Propidium iodide (PI), a DNA binding fluorescent dye, was emitted a red fluorescent light just in dead cells. (McGoverin et al. 2020). Consistent with the results above and compared with the control group, CLSM images of biofilm inhibition showed less biomass by CERI treatment (Fig. 1E), and the images of biofilm eradication exhibited more dead bacterial cells by CERI treatment (Fig. 1F). Likewise, fluorescence intensity analysis of CLSM images showed that the fluorescent quantitation of CERI was reduced sharply to almost a quintile of the control in inhibition (Fig. 1G). For eradication, the fluorescent quantitation was nearly a quarter of that of the untreated cells (Fig. 1H). These results illustrated that the therapeutic effect of CERI should not be underestimated in biofilm-related disease.

### CERI disrupts the cell membrane integrity of *S. aureus* and induces reactive oxygen species (ROS) production

To detect the cell membrane-disrupting activity of CERI, we first stained CERI-treated bacterial cells with the membrane permeability-sensitive fluorescent dye SYTOX Green. As expected, CERI showed significant cell membrane-disrupting activity against *S. aureus* with a concentration-dependent increasement in the fluorescence intensity of ~ 1000 AU at 32 μg/mL (Additional file 3: Fig. S2A). Similarly, as visualized by CLSM observation, the majority of bacterium in the bright field and fluorescence channels largely overlapped, indicating that the fluorescent dye penetrated the membrane-broken cells (Additional file 3: Fig. S2B). Next, we used the fluorescent dye DiSC3(5), a membrane potassium ion-sensitive probe that accurately and sensitively reflects changes in membrane potential, to detect the cell membrane-disrupting activity of CERI. Similarly, as the positive control melittin increased the fluorescence intensity by ~ 300 AU, treatment with 8 μg/mL CERI against *S. aureus* ATCC 43300 enhanced the fluorescence intensity almost like melittin in a concentration dependent manner (Additional file 3: Fig. S2C). Then, the membrane permeability of the *S. aureus* strain was further evaluated by PI staining, which indirectly determined the permeability of the cell membrane. As we expected, the fluorescence intensity was largely increased in the presence of CERI at a concentration of 1 × MIC in a dose-dependent manner. (Additional file 3: Fig. S2D).

Fluidity represents the flowing and rotating property of the lipids in the membrane (Gunther et al. 2021). We used Laurdan dye, a fluorescent probe designed for studying dipolar relaxation and membrane heterogeneity, to detect membrane fluidity after treatment by CERI. As shown in Additional file 3: Fig. S2E, compared with cell membrane-targeting antibiotics such as daptomycin and melittin, the GP value of CERI was significantly decreased at a concentration of 32 μg/mL, which further confirmed that the mechanism of CERI was mediated by targeting the bacterial cell membrane. We further explored the effects of CERI on lipid bilayers by using fluorescently labeled GUVs consisting of DOPC/DOPG (7:3). Lipids collapsed on the surfaces of GUVs at a CERI concentration of 32 μg/mL, which further demonstrated that CERI disrupted the bacterial lipid bilayer of the cell membrane (Additional file 3: Fig. S2F).

It was universally acknowledged that oxidative damage, especially ROS, has a destructive effect on the function of proteins, played a prominent role in metabolic disturbance, and may even result in bacterial death (Ezraty et al. 2017). Hence, we measured the ROS level by using the indicator H2DCFDA (Shi et al. 2021) and found that CERI could significantly elevate the ROS level in bacteria at a concentration of 8 μg/mL (Additional file 3: Fig. S2G). Taken together, CERI kills *S. aureus* cells, probably by disrupting cell membrane permeability, causing membrane depolarization and enhancing ROS production.

### Observation of membrane disruption of CERI by SEM and TEM

**Fig. 2:**
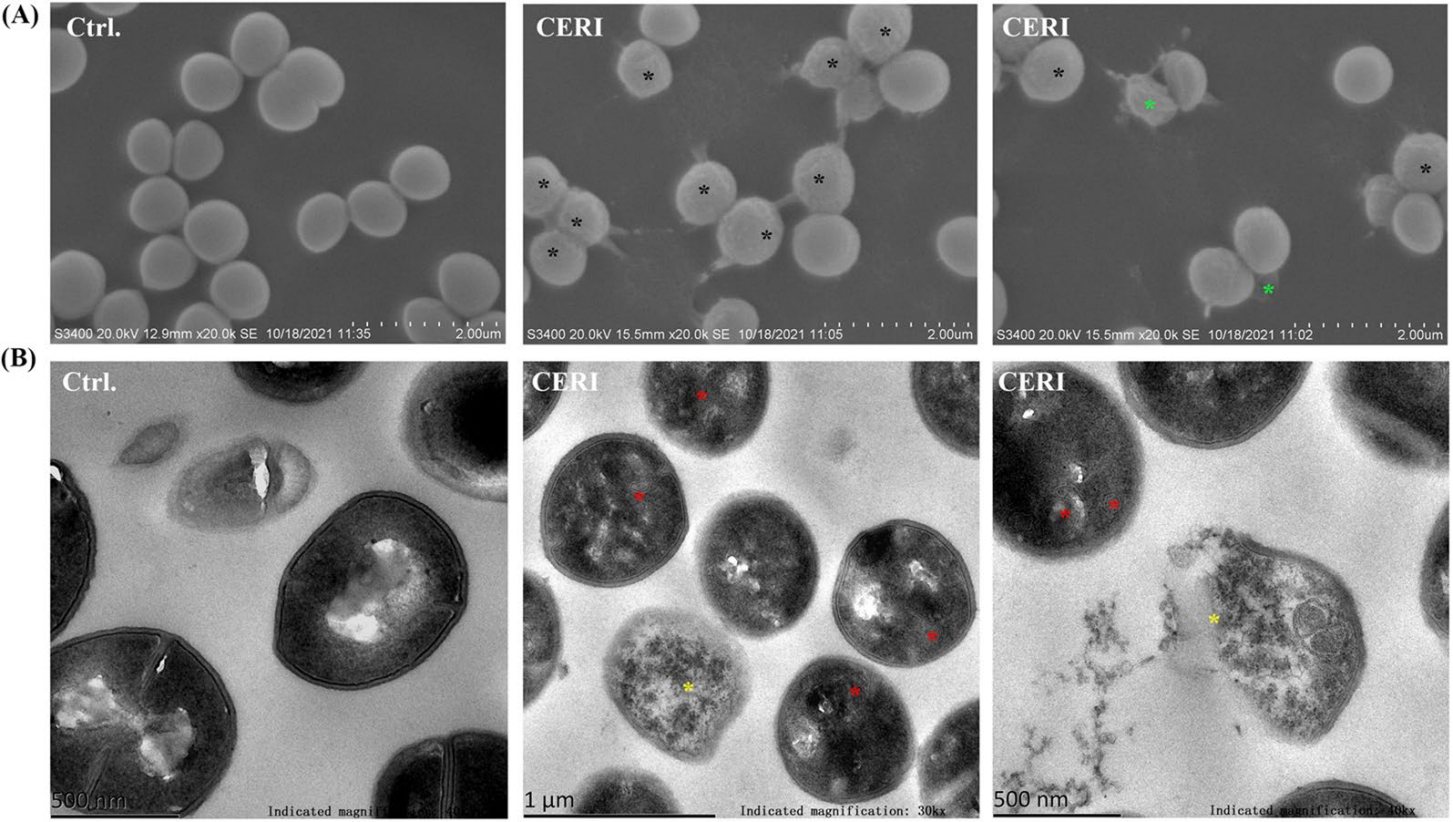
Membrane disruption of CERI by SEM and TEM observation. *S. aureus* ATCC 43300 was treated with 32 μg/mL CERI for 1 h. **A** SEM showed shrinkage (black star) and collapse in the terminal stage (green star). Scale bars, 2 μm. **B** TEM showed mesosome-like structures (red star), cell membrane damage and cell lysis (yellow star). Scale bars, 500 nm (left and right) and 1 μm (middle)

To intuitively observe the membrane disruption caused by CERI, SEM and TEM were used for further study. SEM images showed the shrinkage, detachment and collapse of *S. aureus* strains in the CERI-treated group, and leakage of the intracellular mass could be seen, but the membrane was clear, and ultrastructural changes were not observed in the untreated group (Fig. 2A). In addition, TEM images showed a series of pathological changes in cells, including the formation of mesosome-like structures, cell membrane damage, plasmolysis, disruption of cytoplasm and cell lysis in the presence of 2 × MIC CERI (Fig. 2B).

### Bactericidal activity of CERI against *S. aureus* persisters

**Fig. 3:**
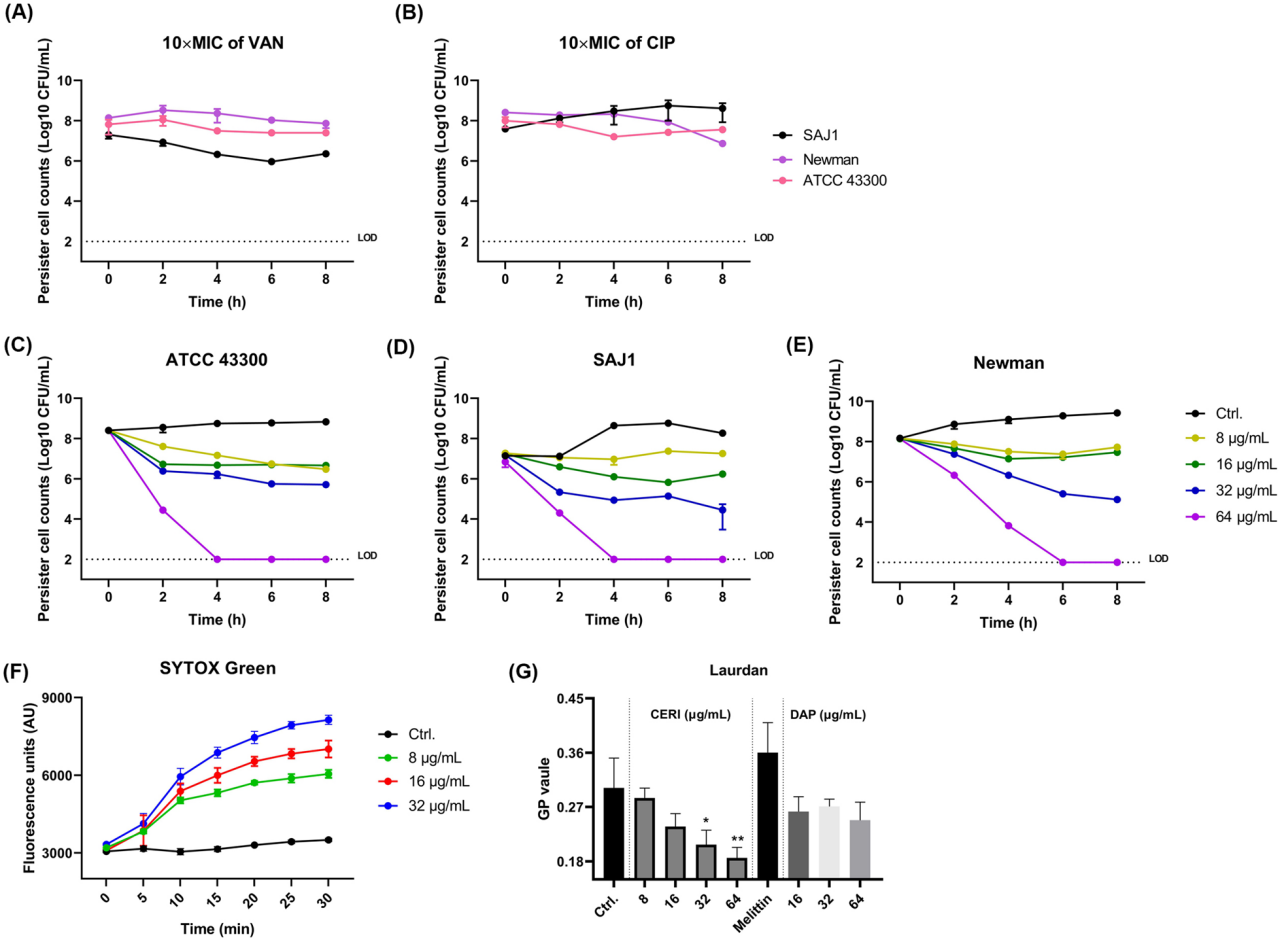
The effects of CERI against *S. aureus* persisters. High resistance of *S. aureus* persisters against 10 × MIC of vancomycin (**A**) and ciprofloxacin (**B**). Time-killing activities of CERI against persisters of MRSA strain ATCC 43300 (**C**), VISA strain SAJ1 (**D**) and MSSA strain Newman (**E**) at concentrations of 16–128 μg/mL (1–8 × MIC). Persister cell membrane permeability was determined by SYTOX Green staining after treatment with various concentrations of CERI. (G) Detection of intracellular and extracellular ATP in *S. aureus* ATCC 43300 after treatment with 8–32 μg/mL of CERI for 30 min. The assays were repeated three times independently, and the results were presented as mean ± SD. Results were considered significant when *P < 0.05 and highly significant when **P < 0.01

It’s reported that persisters are a transient phenotype in bacterial cultures with non-growing state and tolerance to lethal concentrations of antibiotics (Peyrusson et al. 2020). To confirm the formation of persisters rather than metabolically active cells, three preformed persister isolates displayed a high level of tolerance to 10 × MIC of several conventional antibiotics, including VAN and CIP (Fig. 3A and B). Furthermore, we conducted a persister killing assay, and CERI showed effective bactericidal effects against the MRSA strain ATCC 43300 (Fig. 3C) and the VAN-intermediate *S. aureus* strain SAJ1 (Fig. 3D) at a concentration of 1 × MIC in a dose-dependent manner. CERI could also kill all viable persisters at a concentration of 8 × MIC within 4 h. However, the persisters formed by the MSSA Newman strain showed relatively higher resistance to CERI treatment. CERI started to kill its persisters at a concentration of 4 × MIC, and it took 6 h to eliminate all persister cells at a concentration of 8 × MIC (Fig. 3E).

To further explore the mechanism of the persistent killing effects of CERI, we performed a SYTOX Green staining assay. As shown in Fig. 3F, the fluorescence intensity increased rapidly within 30 min in the presence of various concentrations of CERI. Hence, the bacterial cell membrane disrupting activity of CERI still played an important role in its persister killing activity. Moreover, CERI could also increase the intracellular and extracellular ATP of the persister cells, which indicates that CERI may transfer the dormant status of the persisters into the metabolically active status with more susceptibility to CERI treatment (Fig. 3G).

### CERI showed acceptable safety in mammalian cells

Based on the human hemolysis assay, we observed that CERI showed little hemocytolysis at bactericidal concentrations up to 32 μg/mL (Additional file 4: Fig. S3A). In addition, there was no decline in the viability of HK-2 cells (a normal human proximal tubular cell line), even up to a concentration of 128 μg/mL (Additional file 4: Fig. S3B). However, 786-O, a renal cancer cell line, showed an extremely low survival rate with a viability less than 30% in the presence of CERI at the tested concentrations (Additional file 4: Fig. S3C). In summary, the low hemolysis and selective cancer cell inhibition activity of CERI favored its clinical application.

### CERI ameliorates infection in subcutaneous abscesses mouse model

**Fig. 4:**
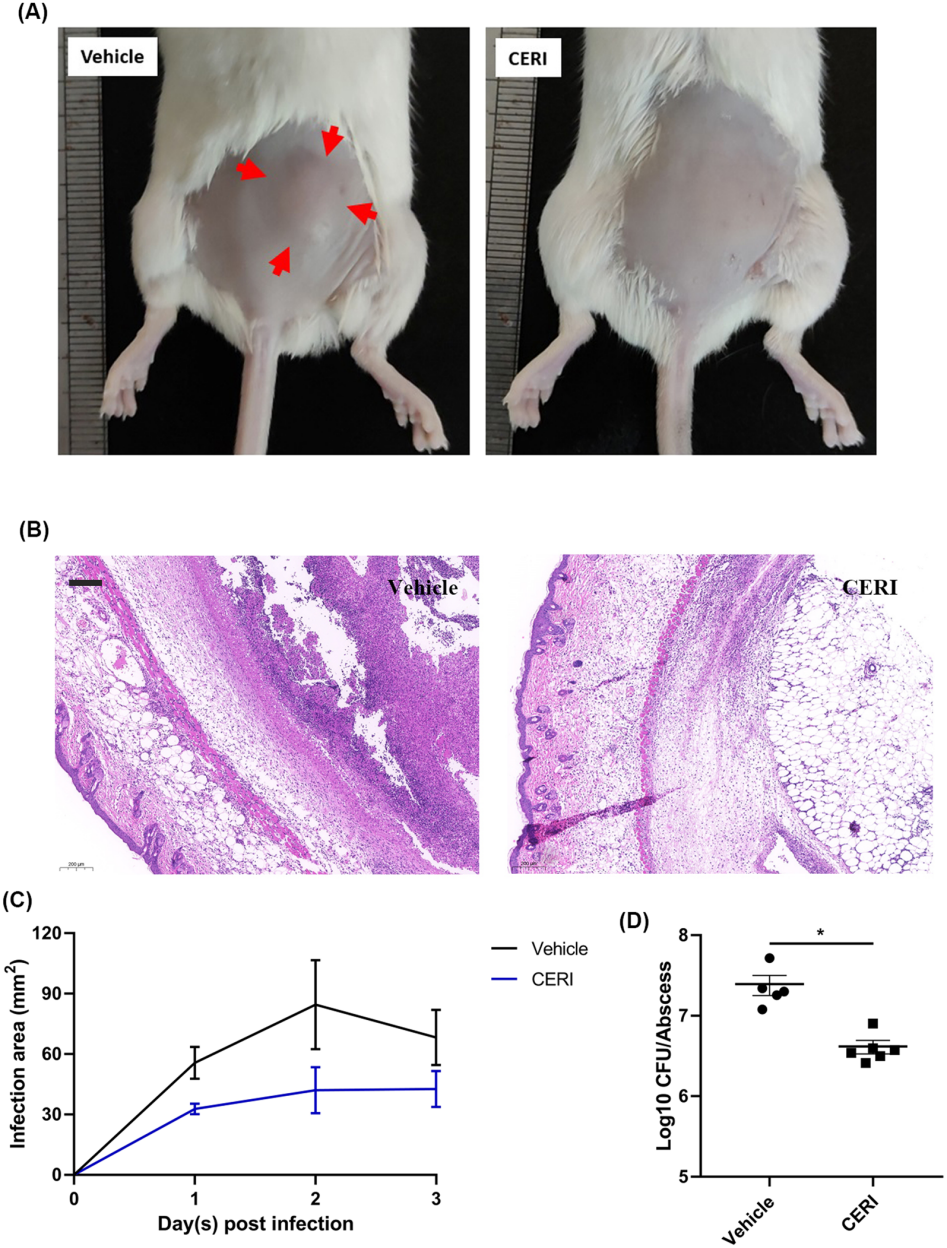
The in vivo antimicrobial activity of CERI in a subcutaneous abscess mouse model. **A** Photographs of subcutaneous abscesses in the vehicle- and CERI-treated groups on the third day post-treatment. Red arrows indicate the boundary of the abscess. **B** H&E staining of abscess tissues treated with vehicle or CERI. Scale bars, 200 μm. **C** Abscess area quantification post infection. **D** Bacterial load quantification in the abscess. Each group has six mice. The assays were repeated three times independently, and the results were presented as mean ± SD. Results were considered significant when *P < 0.05

As shown in Fig. 4A, we observed that CERI distinctly diminished the area of abscess compared with that in the vehicle group 3 days post-infection with *S. aureus* ATCC 43300. The H&E straining of the cutaneous abscess area apparently displayed an inflammatory response with multiple inflammatory cell infiltration and tissue turgor, and even dermo-necrosis in the vehicle group, while the group treated with CERI exhibited less minimal detectable invasion and more normal structures (Fig. 4B). Accordingly, the quantification of the abscess area showed significant inflammation inhibition activity by CERI (Fig. 4C). In addition, the viable bacterial loads in the CERI-treated group were significantly lower than those in the vehicle group (Fig. 4D).

### CERI represents negligible systemic toxicity in vivo

**Fig. 5:**
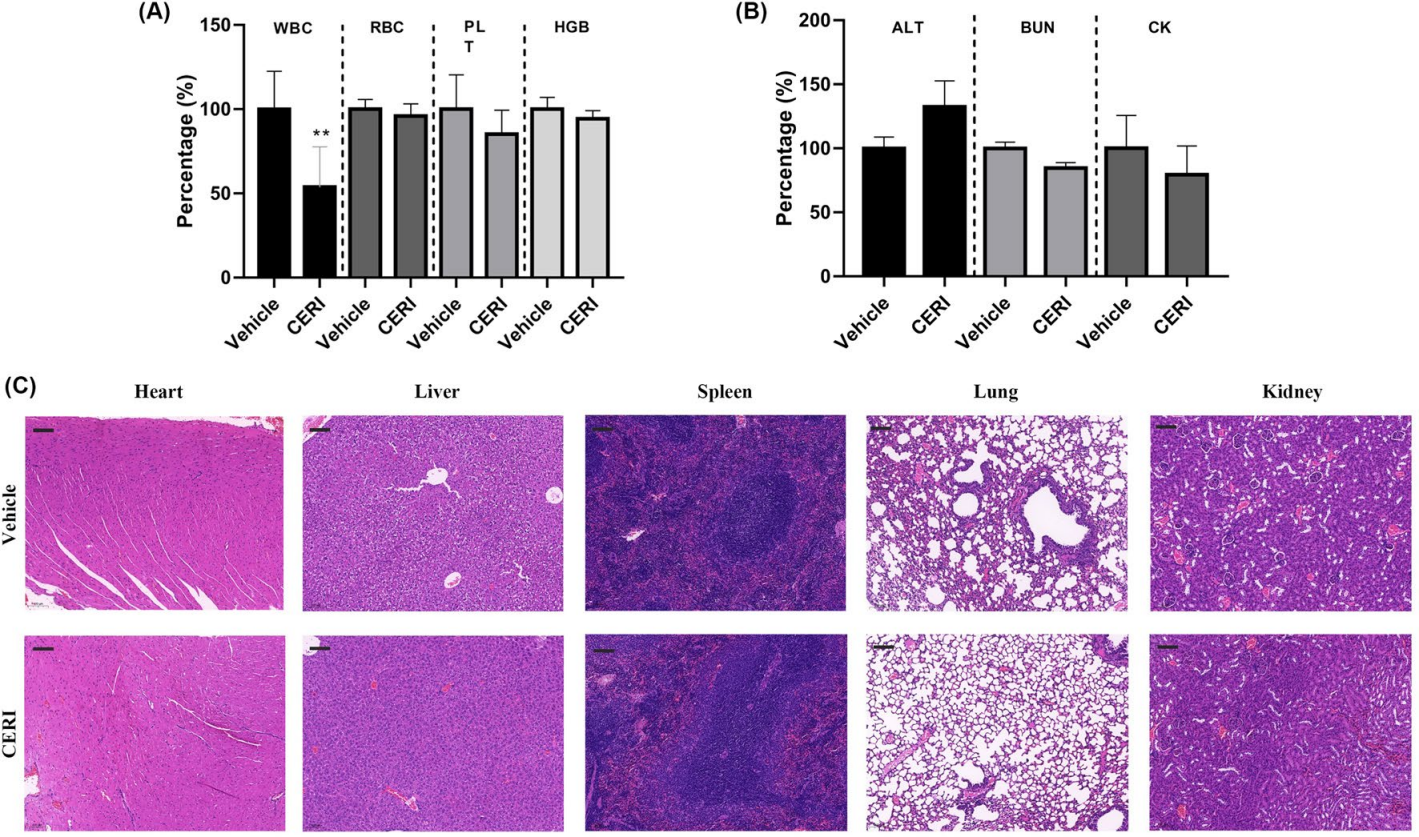
Toxicological evaluation in vivo. Routine blood (**A**) and serum biochemistry examinations (**B**) were performed in the vehicle- and CERI-treated groups. WBC, white blood cell. RBC, red blood cell. PLT, platelet. HGB, hemoglobin. ALT, alanine transaminase. BUN, blood urea nitrogen. CK, creatine kinase. **C** Comparison of pathological changes in organ structure (heart, liver, spleen, lung, kidney) between the vehicle and CERI groups by H&E staining. Scale bars, 200 μm

In its dosage as an antimicrobial agent, we observed that the CERI-treated group showed significantly lower WBC counts than the vehicle group, which could be due to its ability to suppress the release of neutrophils (Guisier et al. 2020). However, we found no indiscriminate variation in RBC, PLT, or HGB (Fig. 5A), providing direct evidence that CERI was almost harmless to blood components at the tested dosage. Similarly, as shown in Fig. 5B, there were no statistically significant differences between the two groups for ALT, BUN, and CK detection, indicating that CERI had no detectable toxicity to the liver, kidney, or heart at the tested dosage. Furthermore, the H&E staining displayed in Fig. 5C shows there were no visible pathological changes in the tested organs of either the CERI-treated group or the vehicle group. Overall, CERI is safe for normal mammalian cells and has low in vivo systemic toxicity as an antimicrobial agent.

## Discussion

*Staphylococcus aureus* is a common pathogen that causes a wide range of infectious diseases (Tong et al. 2015). In this article, the results indicate that CERI, an antineoplastic drug, was ineffective against gram-negative bacteria but had strong antimicrobial activity against gram-positive bacteria, especially *S. aureus*. Bacterial membrane is crucial to maintain cell integrity that contribute to substance transportation and cellular communication (García-Fernández et al. 2017; Kosgodage et al. 2019; Strahl and Errington 2017). For this reason, the bacterial cytoplasmic membrane is principally targeted by many conventional antibiotics. Through our research, several fluorescent dyes (like SYTOX Green, DiSC3(5) and PI) were used to judge the degree of bacterial membrane damage. Besides, the usage of Laurdan dye further verifies that CERI promotes bacterial cell membrane heterogeneity and structural rearrangement. The present data that indicate a common conclusion: CERI depolarizes the membrane potential and disrupts the membrane integrity of *S. aureus*. Moreover, we attempted to explore the interaction of CERI and metabolism. Fluorescence probe H2DCFDA was used for ROS detection in the bacteria and CERI was observed can induce a rapid rise in the intracellular levels of ROS. Therefore, inducing ROS production in *S. aureus* was another reason for antibacterial effect of CERI. However, as a tyrosine kinase inhibitor, the antibacterial mechanism of CERI hasn’t discussed broodingly and thoroughly in signal pathway of bacteria. That is because tyrosine kinases encoded by *S. aureus* share no structural similarity with their eukaryotic counterparts (Fukazawa et al. 2019). Two tyrosine kinases (CapB1 and CapB2) have been identified in the *S. aureus* genome. In earlier studies, there was no evidence that CapB1 had kinase activity in purified enzymes (Gruszczyk et al. 2013).

In addition, the planktonic cell state is considered more sensitive to antimicrobial chemicals and the human immune response system; therefore, conferring the ability of biofilm eradication is an extra bonus of antibiotics (Rumbaugh and Sauer 2020). In this study, CERI was found to be highly effective in inhibiting biofilm formation and dispersing biofilms formed by *S. aureus*. Moreover, persister cells attract much attention from the global public because of their obstinate survivability to antimicrobial agents and pursuit of loss in clinical treatments (Khan et al. 2020). CERI showed direct inhibition and fierce lethality to the persister cells of the *S. aureus* strain. In addition, it also increased ATP levels and interferes with the energy metabolism of persisters. The elimination of biofilms and persisters further promotes the clinical application of CERI.

*Staphylococcus aureus* skin and soft tissue infections possess a high recurrence rate, and are the most common clinical symptoms from minor ailments to life threatening infections Vella et al. (2021). In our subcutaneous abscess mouse model, CERI showed the ability to restrict the area of abscess, reduce bacterial loads, and eminently exhibit antimicrobial and anti-inflammatory effects in vivo. In the basis of pharmacokinetic properties studied before, the peak concentrations of CERI were achieved within 6 h accounting for 97% of CERI was bounded to human plasma proteins with concentration independence. The mean half-life period of CERI was approximately 40 h and exhibited non-linear pharmacokinetics over time (Hirota et al. 2019; Shaw et al. 2014).

In earlier research, assessment and prediction to safety of CERI are established on the base of experiments. Gastrointestinal function abnormal are the most common side effects of CERI. Asian patients with non-small cell lung cancer treated with CERI at a dose of 450-mg fed showed consistent efficacy and less gastrointestinal toxicity than 750-mg fasted patients (Cho et al. 2019). In vitro safety assessment, we observed a slight hemolysis phenomenon, reminding us to pay more attention to the recommended dosage. Besides, we also found that 786-O inhibited growth and the viability dropped sharply. This phenomenon suggested that CERI had an enormous effect on repressing cancer cells according to the original antineoplastic purpose. Fortunately, CERI shows brilliant antibacterial efficiency at very lower concentration than mentioned above, which will not limit the clinical application and popularization of drugs. And beyond that, there is not currently any data on carcinogenicity, effect on human fertility, or on early embryonic development (Khozin et al. 2015).

There is no article reporting potent anti-staphylococcal activity of CERI; in other words, this is the first study on CERI-eliminated biofilms and the persister bacteria of *S. aureus,* and it shows that the leading mechanism of CERI retards bacteria. In conclusion, these discussions elucidate that CERI possesses tremendous research and application potential to solve the dilemma of chronic and recurrent infections produced by biofilms and persisters, and are expected to be an alternative to CIP, VAN, and RFP when the microorganisms become resistant to those antimicrobials.

CERI appears to have tremendous potential, however there are still some deficiencies in our study that could be improved upon. First, we chose an abscess mouse animal as our model, but it only reflects the cutaneous colonization of *S. aureus,* and the arguments were inadequate to evaluate the efficacy in other sites of infection. Furthermore, CERI does not allow for long-term usage because of non-negligible cytotoxicity. In addition, the mechanism of biofilm eradication is not clear and is worth further discussion in future studies.

In summary, our results warrant further development of CERI as a potential therapeutic for intractable infectious diseases caused by antibiotic-resistant or persistent *S. aureus*.

## Supplementary Information

**Additional file 1: Table S1.** Structure and MICs (against *S. aureus* ATCC 43300) of the selected 6 hits.

**Additional file 2: Figure S1.** Antimicrobial effects of CERI against *S. aureus* ATCC 43300 planktonic cells. (A) The flow chart of high throughput screen for repurposing ceritinib. (B) The chemical structure formula of CERI. Time-dependent bacteriostatic (C) and bactericidal (D) effects of CERI against S. aureus ATCC 43300. (E) The plate images for the antimicrobial activity against *S. aureus* ATCC 43300 in vitro.

**Additional file 3: Figure S2.** Antibacterial mechanisms of CERI against *S. aureus*. (A) *S. aureus* ATCC 43300 was treated with CERI (16 μg/mL), melittin (16 μg/mL) and DMSO, and the fluorescence intensity of SYTOX Green was measured within 25 min. (B) SYTOX Green staining visualization by CLSM. (C) The fluorescence intensity of DiSC3(5) was determined by CERI (16 μg/mL), melittin (16 μg/mL) and DMSO treatment. (D) PI staining was performed by CERI treatment for 30 min. (E) Membrane fluidity was evaluated based on the generalized polarization (GP) index after treatment with the indicated concentrations of CERI. (F) GUVs labeled with FITC were treated with 32 μg/mL CERI or 0.1% DMSO (control) for 15 min and were captured by CLSM. Scale bars, 20 μm. (G) Quantification of ROS release after treatment with CERI. The assays were repeated three times independently, and the results were presented as mean ± SD. Results were considered significant when *P < 0.05 and highly significant when **P < 0.01 and ***P < 0.001.

**Additional file 4: Figure S3.** Hemolysis and cytotoxicity of CERI. (A) Hemolysis of human red blood cells treated with 1–32 μg/mL CERI. Triton X-100 was used as a positive control. DMSO (0.2%) was used as a negative control. Cell viability of HK-2 (B) and 786-O (C) cells in the presence of the indicated concentrations of CERI.

## Data Availability

The datasets used and/or analyzed during the current study are available from the corresponding author on reasonable request.

## Abbreviations

CERI: Ceritinib
MRSA: Methicillin-resistant *Staphylococcus aureus*
MSSA: Methicillin-susceptible *Staphylococcus aureus*
ALK: Anaplastic lymphoma kinase
FDA: Food and Drug Administration
BHI: Brain–heart infusion
TSB: Soybean trypsin broth
LB: Luria–Bertani broth
CLSI: Clinical and Laboratory Standards Institute
MH: Mueller–Hinton broth
MIC: Minimum inhibitory concentration
MBC: Minimum bactericidal concentration
RFP: Rifampicin
CIP: Ciprofloxacin
VAN: Vancomycin
DMSO: Dimethyl sulfoxide
CFUs: Colony-forming units
CV: Crystal violet
OD: Optical density
H&E: Hematoxylin and eosin
RBCs: Red blood cells
