# Supplementary material for: Insights into the antimicrobial effects of ceritinib against Staphylococcus aureus in vitro and in vivo by cell membrane disruption
Source: AMB Express. 2022 Nov 28;12:150. doi: 10.1186/s13568-022-01492-w (PMC9705652; doi:10.1186/s13568-022-01492-w)
Supplement: Supplementary file 1 — Additional file 1: Table S1. Structure and MICs (against S. aureus ATCC 43300) of the selected 6 hits. [file 13568_2022_1492_MOESM1_ESM.docx]

Table S1. Structure and MICs (against *S. aureus* ATCC 43300) of the selected 6 hits.

| Drugs | Structure | MIC (μg/mL) |
| --- | --- | --- |
| Astemizole | 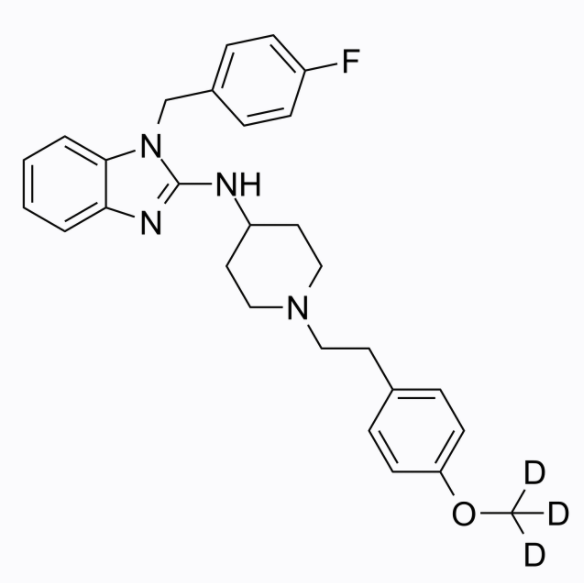 | 32 |
| Fluralaner | 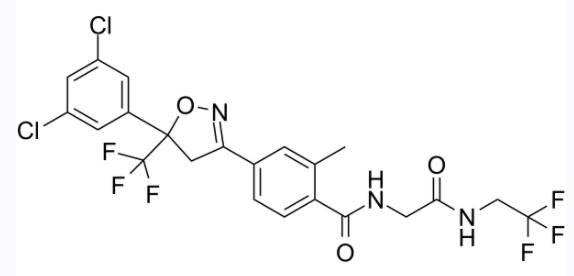 | 32 |
| Asunaprevir | 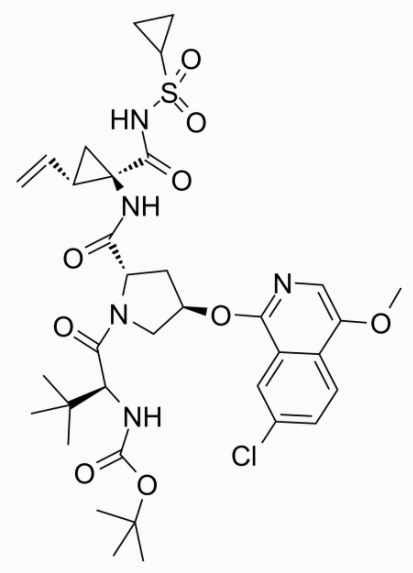 | 32 |
| Voxilaprevir | 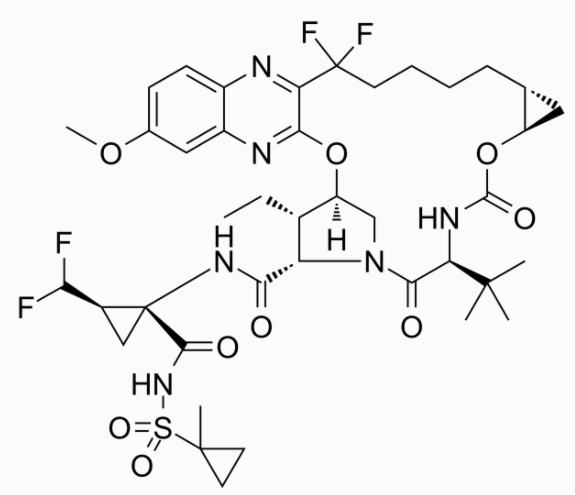 | 64 |
| Daprodustat | 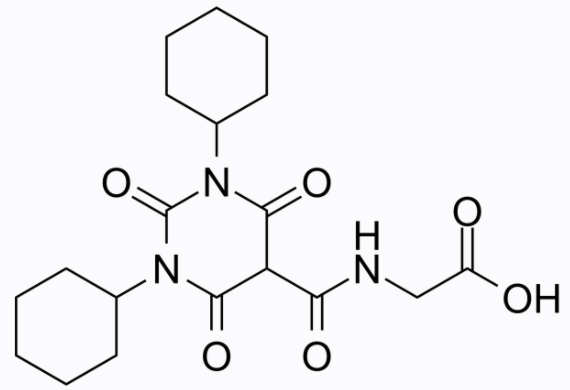 | 32 |
| CERI | 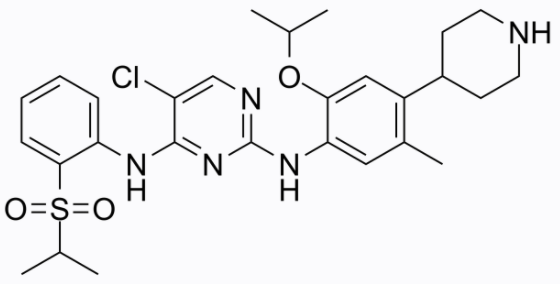 | 16 |
