# Supplementary figures and images for: Insights into the antimicrobial effects of ceritinib against Staphylococcus aureus in vitro and in vivo by cell membrane disruption
Source: AMB Express. 2022 Nov 28;12:150. doi: 10.1186/s13568-022-01492-w (PMC9705652; doi:10.1186/s13568-022-01492-w)

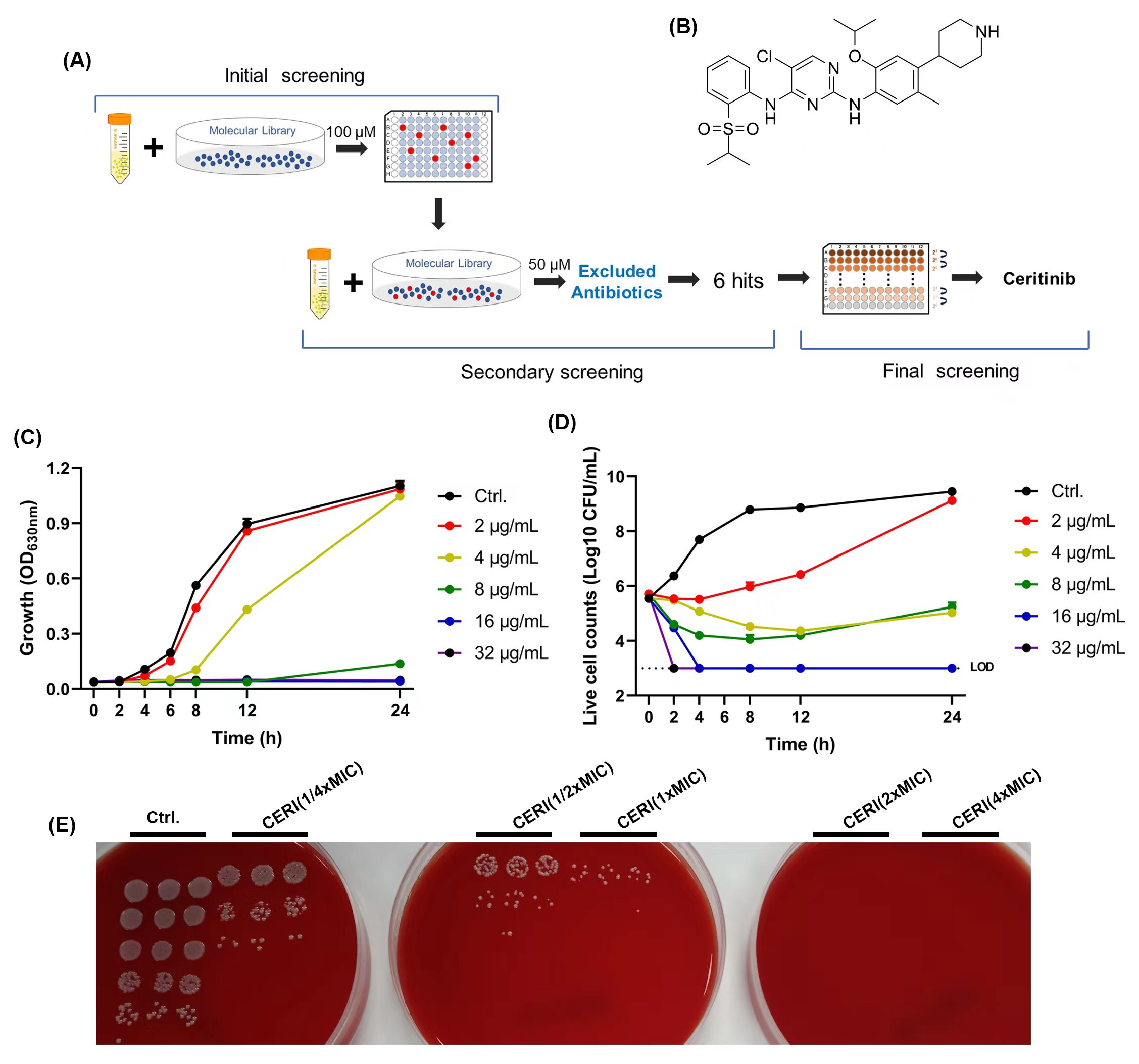

Supplement: Supplementary file 2 — Additional file 2: Figure S1. Antimicrobial effects of CERI against S. aureus ATCC 43300 planktonic cells. (A) The flow chart of high throughput screen for repurposing ceritinib. (B) The chemical structure formula of CERI. Time-dependent bacteriostatic (C) and bactericidal (D) effects of CERI against S. aureus ATCC 43300. (E) The plate images for the antimicrobial activity against S. aureus ATCC 43300 in vitro. [file 13568_2022_1492_MOESM2_ESM.jpg]

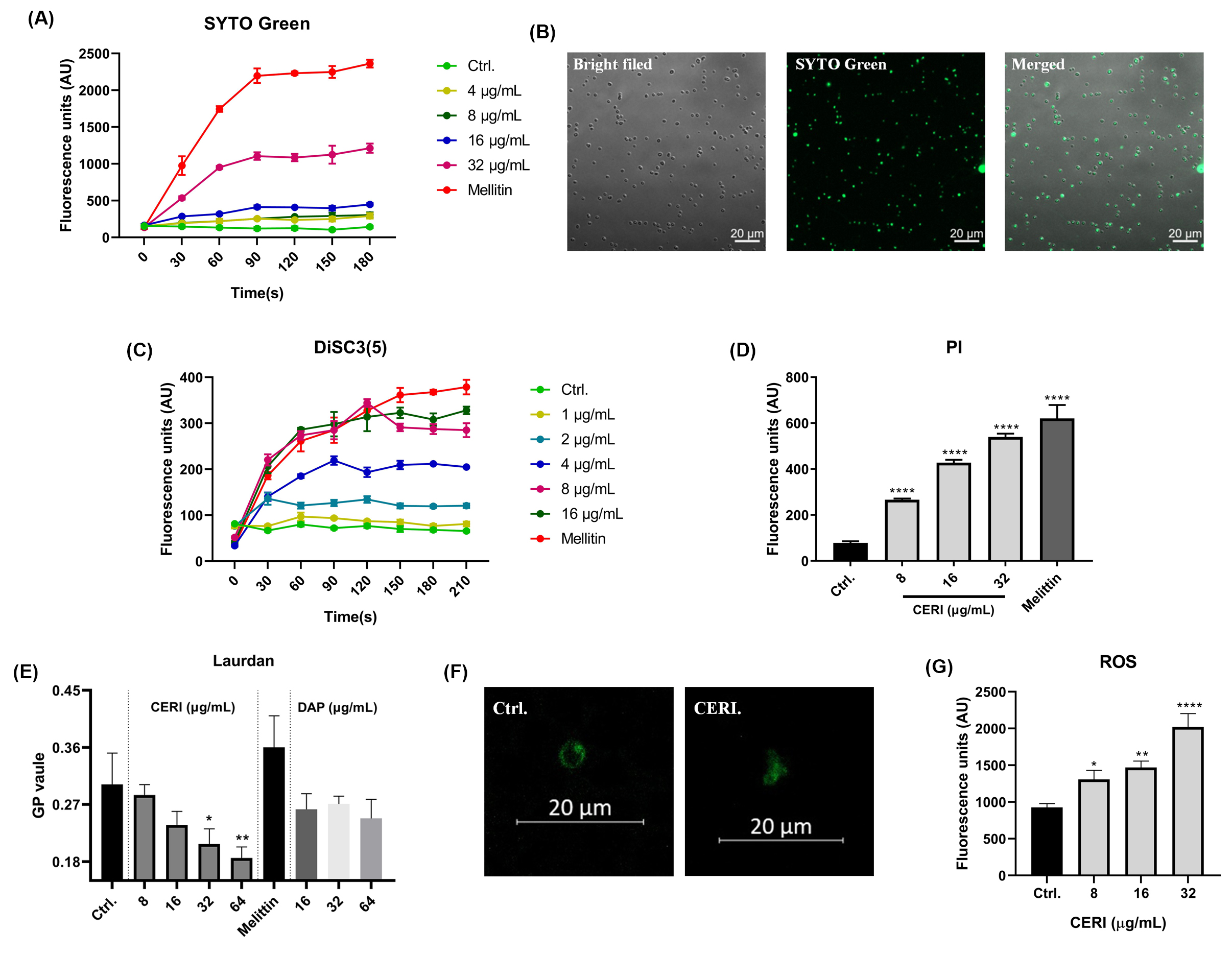

Supplement: Supplementary file 3 — Additional file 3: Figure S2. Antibacterial mechanisms of CERI against S. aureus. (A) S. aureus ATCC 43300 was treated with CERI (16 μg/mL), melittin (16 μg/mL) and DMSO, and the fluorescence intensity of SYTOX Green was measured within 25 min. (B) SYTOX Green staining visualization by CLSM. (C) The fluorescence intensity of DiSC3(5) was determined by CERI (16 μg/mL), melittin (16 μg/mL) and DMSO treatment. (D) PI staining was performed by CERI treatment for 30 min. (E) Membrane fluidity was evaluated based on the generalized polarization (GP) index after treatment with the indicated concentrations of CERI. (F) GUVs labeled with FITC were treated with 32 μg/mL CERI or 0.1% DMSO (control) for 15 min and were captured by CLSM. Scale bars, 20 μm. (G) Quantification of ROS release after treatment with CERI. The assays were repeated three times independently, and the results were presented as mean ± SD. Results were considered significant when *P < 0.05 and highly significant when **P < 0.01 and ***P < 0.001. [file 13568_2022_1492_MOESM3_ESM.jpg]

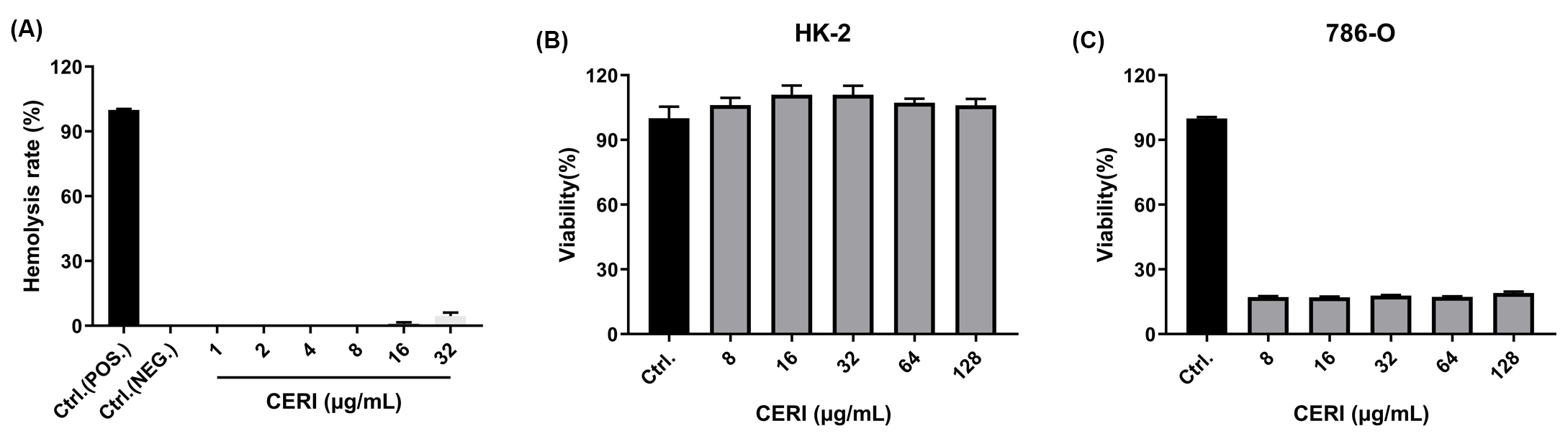

Supplement: Supplementary file 4 — Additional file 4: Figure S3. Hemolysis and cytotoxicity of CERI. (A) Hemolysis of human red blood cells treated with 1–32 μg/mL CERI. Triton X-100 was used as a positive control. DMSO (0.2%) was used as a negative control. Cell viability of HK-2 (B) and 786-O (C) cells in the presence of the indicated concentrations of CERI. [file 13568_2022_1492_MOESM4_ESM.jpg]
